# Investing in a global pooled-funding mechanism for late-stage clinical trials of poverty-related and neglected diseases: an economic evaluation

**DOI:** 10.1136/bmjgh-2023-011842

**Published:** 2023-05-29

**Authors:** Armand Zimmerman, Mohamed Mustafa Diab, Marco Schäferhoff, Kaci Kennedy McDade, Gavin Yamey, Osondu Ogbuoji

**Affiliations:** 1 Center for Policy Impact in Global Health, Duke Global Health Institute, Duke University, Durham, North Carolina, USA; 2 Open Consultants, Berlin, Germany

**Keywords:** Health economics, Health policy, Infections, diseases, disorders, injuries

## Abstract

**Introduction:**

Poverty-related and neglected diseases (PRNDs) cause over three million deaths annually. Despite this burden, there is a large gap between actual funding for PRND research and development (R&D) and the funding needed to launch PRND products from the R&D pipeline. This study provides an economic evaluation of a theoretical global pooled-funding mechanism to finance late-stage clinical trials of PRND products.

**Methods:**

We modelled three pooled-funding design options, each based on a different level of coverage of candidate products for WHO’s list of PRNDs: (1) vaccines covering 4 PRNDs, (2) vaccines and therapeutics covering 9 PRNDs and (3) vaccines, therapeutics and diagnostics covering 30 PRNDs. For each option, we constructed a discrete event simulation of the 2019 PRND R&D pipeline to estimate required funding for phase III trials and expected product launches through 2035. For each launch, we estimated global PRND treatment costs averted, deaths averted and disability-adjusted life-years (DALYs) averted. For each design option, we calculated the cost per death averted, cost per DALY averted, the benefit–cost ratio (BCR) and the incremental cost-effectiveness ratio (ICER).

**Results:**

Option 1 averts 18.4 million deaths and 516 million DALYs, has a cost per DALY averted of US$84 and yields a BCR of 5.53. Option 2 averts 22.9 million deaths and 674 million DALYs, has a cost per DALY averted of US$75, an ICER over option 1 of US$49 and yields a BCR of 3.88. Option 3 averts 26.9 million deaths and 1 billion DALYs, has a cost per DALY averted of US$114, an ICER over option 2 of US$186 and yields a BCR of 2.52.

**Conclusions:**

All 3 options for a pooled-funding mechanism—vaccines for 4 PRNDs, vaccines and therapeutics for 9 PRNDs, and vaccines, therapeutics and diagnostics for 30 PRNDs—would generate a large return on investment, avert a substantial proportion of the global burden of morbidity and mortality for diseases of poverty and be cost-effective.

**WHAT IS ALREADY KNOWN ON THIS TOPIC**

**WHAT THIS STUDY ADDS**

- Our study estimates the potential impact of a global pooled-funding mechanism to finance late-stage clinical trials of PRND products. Our results show that investments in late-stage clinical trials for PRNDs may avert up to 26 million deaths and 1 billion disability-adjusted life-years globally over the period 2019–2035, with economic returns to society that outweigh the costs of investment.

**HOW THIS STUDY MIGHT AFFECT RESEARCH, PRACTICE OR POLICY**

- This study highlights the value of a new financial model to increase coordination and collaboration across R&D initiatives for PRNDs, to mobilise new funding sources for PRND product development, to reduce the financial risk associated with PRND investments and to curate a global portfolio of ideal PRND product investment opportunities. Such a model may avert millions of deaths and billions in treatment costs worldwide.

## Introduction

 Poverty-related and neglected diseases (PRNDs) are a major contributor to the disease burden in low-income and middle-income countries (LMICs).^1^ These conditions are classified by the WHO as type II diseases that disproportionately affect LMICs or type III diseases that are prevalent exclusively in LMICs.^2^ PRNDs include HIV, tuberculosis (TB), malaria, pneumonia, diarrhoeal diseases and all other neglected tropical diseases (NTDs).^3^ In 2019, HIV caused 864 thousand deaths globally, TB 1.18 million, malaria 643 thousand, pneumonia 2.49 million, diarrhoeal diseases 1.53 million and all other NTDs 104 thousand.^4^ Over 90% of these PRND-related deaths occurred in LMICs.^4^

Investments in PRND product research and development (R&D), especially for late-stage clinical trials, are not commensurate with the global burden of disease caused by PRNDs. PRNDs account for 13.8% of the global disease burden, yet receive 1.34% of the world’s total health R&D expenditure.^5^ According to the 2019 Global Funding of Innovation for Neglected Diseases (G-FINDER) survey, global R&D funding for PRND product development reached US$4.1 billion in 2018.^6^ However, there is a major gap between 2018 investments and the amount needed to launch PRND candidates from the R&D pipeline. For example, an estimated US$16.3 billion was required to launch 128 products from the 2017 R&D pipeline containing 538 PRND product candidates. More than half (55%) of the US$16.3 billion was needed for phase III clinical trials alone.^7^ Yet in 2018, only 35% of the US$4.1 billion dedicated to PRND candidates in the R&D pipeline was spent on clinical development and postregistration studies.^6^ The remaining 65% of funds were allocated towards basic and early-stage research or research for unspecified R&D stages. There is a need to increase overall PRND funding and, more importantly, to close the funding gap between early-stage and late-stage research for PRND products.

There are three main reasons why the PRND funding gap for R&D persists. First, the high cost of phase III clinical trials as described above can make investors reluctant to fund trials for a product/disease with a small market. Second, PRNDs overwhelmingly occur in LMICs, but LMIC markets cannot sustain the costs required to support a PRND product development pipeline that includes vaccines, drugs and diagnostics.^8^ The commercial potential of new PRND technologies in these countries is simply too small to incentivise R&D investments.^9^ Third, there is insufficient funding coordination across PRND R&D initiatives. There is no globalised system for establishing consensus on R&D priorities for PRNDs, no universal portfolio of ideal PRND products to invest in, and a lack of engagement with LMIC partners during global and regional R&D priority setting discussions.^9^ As a result, new PRND product development gets delayed and preventable PRND related morbidity and mortality persists globally.

Ultimately, a new financial model is needed to increase coordination and collaboration across R&D initiatives for PRNDs, to mobilise new funding sources for PRND product development, to reduce the financial risk associated with PRND investments and to curate a global portfolio of ideal PRND product investment opportunities.^1^ In doing so, a new financial model could also support the use of adaptive clinical trials, which introduce efficiencies into the R&D pathway by shortening trial phase times and lowering overall trial costs.^10^ We investigated the need for and the design of such a financial mechanism which, hereafter, we refer to as a ‘pooled-funding mechanism’. In this study, we describe different pooled-funding mechanism design options and assess the benefits and costs of each design.

## Methods

### Pooled-funding mechanism design options

We conducted a qualitative study to assess the demand for, and design options of, a pooled-funding mechanism for PRND product development. Details of this qualitative study are described elsewhere.^11^ In summary, we performed a literature review to assess the design of existing funding mechanisms that fund various aspects of vaccine development, such as the Coalition for Epidemic Preparedness Innovations, Gavi, the Vaccine Alliance and the European and Developing Country Trial Partnership. Then, between September 2019 and August 2020, we conducted 192 key informant interviews (KIIs) with stakeholders from governments, regulatory bodies, multilateral and global organisations, private pharmaceutical industries and academic institutions. The stakeholders represented four high-income countries (USA, UK, Germany and the Netherlands) and four middle-income countries (India, South Africa, China and Kenya). The first round of interviews, with 165 key informants (KIs), took place from September 2019 to May 2020. Between June and August 2020, we interviewed an additional 27 KIs—including a range of potential funders from LMICs and HICs—to get feedback on the initial design options that emerged from the first round of interviews.

Three pooled-funding mechanism design options (table 1) emerged from our qualitative study: (1) a pooled-funding mechanism for late-stage trials of vaccines to control HIV, TB, malaria and pneumococcal pneumonia, (2) a pooled-funding mechanism for late-stage trials of a wider set of products (vaccines and therapeutics) for a wider range of diseases (HIV, TB, malaria, pneumonias and 5 NTDs) and (3) a pooled-funding mechanism for all product types (vaccines, therapeutics and diagnostics) and 30 PRNDs. In this study, we estimate the number of product launches expected from each design option, the funding necessary to achieve these product launches, and the economic and health benefits accrued globally from each product launch.

**Table 1:** Pooled-funding mechanism design options

Design option	Trial phases funded	Product candidates funded	Diseases funded
Option 1	Phase III trials	Vaccines	HIV, TB, malaria and pneumonia
Option 2	Phase III trials	Vaccines, drugs and diagnostics	HIV, TB, malaria, pneumonia, Chagas, schistosomiasis, leishmaniasis, dengue and leprosy
Option 3	Phase III trials	Vaccines, drugs and diagnostics	HIV, TB, malaria, pneumonia, Chagas, schistosomiasis, leishmaniasis, dengue, leprosy, shigellosis, Ebola, hepatitis C, enterotoxigenic *Escherichia coli*, non-typhoidal salmonella, human African trypanosomiasis, onchocerciasis, cholera, hookworm, meningitis, rheumatic fever, diarrhoeal diseases, Buruli ulcer, trachoma, typhoid, paratyphoid, cryptosporidiosis, multiple salmonella infections, hepatitis B, herpes simplex-2, gonorrhoea and chlamydia.

TB, tuberculosis.

### Analysis perspectives

Our analysis assumes two perspectives: a societal perspective and an altruistic investor perspective. The societal perspective quantifies the returns to society for every US$1 invested in the pooled-funding mechanism. The societal perspective includes start-up and annual operational costs associated with the pooled-funding mechanism, health system strengthening investments, phase III trial costs for product candidates and vaccine procurement costs for any vaccines that launch from the R&D pipeline. The societal perspective also includes three health benefits: disability-adjusted life-years (DALYs) averted, cases averted and deaths averted resulting from product candidates that launch from the R&D pipeline. Lastly, the societal perspective includes one economic benefit:treatment costs averted.

The altruistic investor perspective quantifies returns to society for every US$1 that the investors allocate to the pooled-funding mechanism. We use this altruistic investor perspective because the pooled-funding mechanism functions as a non-profit whereby investments are for the benefit of society rather than for personal profit. The altruistic investor perspective also includes start-up and annual operational costs associated with the pooled-funding mechanism as well as health system strengthening investments and phase III trial costs, but does not include vaccine procurement costs. Vaccine procurement costs are assumed to be borne by individual countries or pooled-procurement entities. Benefits in the altruistic investor perspective include DALYs, deaths, cases and treatment costs averted.

### Phase III trial costs and product launches

We developed a discrete event simulation (DES)^12^ using the MATLAB-based simulation tool SimEvents (online supplemental appendix 1). Our DES simulates the movement of product candidates in each pooled-funding mechanism design option through the PRND R&D pipeline. In our DES, each product candidate is treated as an individual entity with a unique set of attributes: product type, target disease, clinical trial phase lead time, clinical trial phase length time, clinical trial phase probability of success, and clinical trial phase cost. Each clinical trial phase (preclinical, phase I, phase II and phase III) is treated as a ‘server’ with an infinite capacity to store entities. The base case inputs for clinical trial length, probability of success and cost in our DES model are presented in table 2. These base case inputs were derived from a previous study that calculated average preclinical, phase I, phase II and phase III trial lengths, success rates, and costs for vaccine, drug, and diagnostic products based on a review of 25 000 product candidates.^13^ The authors of this study also refined and validated their estimates through triangulation with peer-reviewed literature and interviews with R&D stakeholders.^13^ Estimates from this study have also been used in other analyses.^14^

**Table 2:** Base case inputs for our discrete-event simulation

	Vaccine simple	Vaccine complex	New chemical entity simple	New chemical entity complex	Drug repurposed simple	Drug repurposed complex	Biologic simple	Biologic complex	Diagnostic assay development	Diagnostic simple platform development
**Cost (2020 USD millions**)										
Preclinical	6.66	16.63	5.00	10.00	NA	5.00	10.79	21.59	3.00	NA
Phase I	2.25	2.47	2.21	7.44	NA	2.21	2.41	7.65	2.00	100.00
Phase II	13.22	13.88	5.81	6.39	5.81	5.81	7.53	8.28	3.50	3.50
Phase III	111.10	133.32	32.82	36.10	17.61	17.61	54.12	59.53	NA	NA
**Length (years**)										
Preclinical	3.36	3.33	2.49	2.87	NA	NA	3.29	3.24	1.00	NA
Phase I	1.57	1.97	1.80	1.93	NA	NA	1.62	1.49	1.25	NA
Phase II	2.23	3.71	3.38	3.51	2.14	2.14	2.47	4.16	1.33	2.50
Phase III	2.33	3.50	3.18	2.80	2.14	2.14	2.10	3.38	NA	2.00
**Probability of success**										
Preclinical	0.410	0.410	0.650	0.550	NA	0.750	0.750	0.770	0.500	NA
Phase I	0.684	0.500	0.597	0.572	NA	0.585	0.662	0.696	1.000	0.750
Phase II	0.459	0.216	0.388	0.197	0.457	0.457	0.443	0.322	1.000	1.000
Phase III	0.708	0.636	0.691	0.403	0.681	0.681	0.709	0.625	NA	NA

NA, not applicable.

For each pooled-funding mechanism design option, our DES simulates the PRND R&D pipeline over a 12-year period from 2019 to 2030. Each simulation begins with a portfolio of PRND product candidates that reflects the pooled-funding mechanism design option, and that is based on the actual global portfolio of PRND product candidates as of 31 August 2019. In each simulation, product candidates, therefore, enter the pipeline at the clinical trial phase they were in as of 31 August 2019. Product candidates that were already in phase III trials as of 31 August 2019 were excluded from our simulations because we assumed phase III trials for these candidates were already funded. Online supplemental appendix 1 shows the portfolio of products simulated for each design option.

In addition to the product candidate portfolios simulated with our DES, for each simulation, we assumed 10 new candidates would enter the preclinical trial phase per entity type per year for the first 5 years of the simulation. We also defined a product launch as occurring when a product successfully exits a phase III trial, and to account for the various market entry requirements that might be imposed by different governments, we assumed that each launched product would enter the global market 1 year after launch.^15 16^ For each design option, we ran 100 Monte Carlo simulations. Product launches were averaged across each of the 100 simulations to obtain mean launches per entity type per year. Finally, we validated our model against an excel-based financial forecasting tool called the Portfolio-To-Impact tool that was developed by the Special Programme for Research and Training in Tropical Diseases.^14^

### Economic and health benefits of product launches

For each product launch, we estimated the DALYs, deaths, cases and treatment costs averted that would accrue between the year of market entry and 2035. The calculation of all benefits was based on our assumptions regarding the impact of new vaccines, therapeutics and diagnostics that enter the global market. We assumed that, on market entry, a new vaccine would provide a 10 percent reduction in the annual incidence of disease in the first year, and an additional 10 percent reduction each subsequent year with no more than a 90 percent reduction. Similarly, we assumed that, on market entry, therapeutics and diagnostics would increase treatment coverage by 10 percent in the first year and by an additional 10 percent each subsequent year with no more than a maximum attainment of 90% treatment coverage. These assumptions have been used in previous analyses and are based on increments in intervention coverage thought to be achievable over a 1-year period.^17^ Thus, benefits of the pooled-funding mechanism were based on the difference between burden of disease in a base case scenario and burden of disease in a scenario in which a new product (vaccine, therapeutic and/or diagnostic) is introduced to the global population.

All inputs used to calculate DALYs, years of life lost to premature death (YLLs), years of healthy life lost to disability (YLDs), deaths, cases and treatment costs in the base case scenarios were collected through a review of literature and can be found in online supplemental appendix 1. Inputs needed to inform these calculations included annual incidence, annual prevalence, annual deaths, disability weights, treatment coverage and treatment costs for each disease modelled. Equations 1-9 show how we calculated YLLs, YLDs, DALYs averted, deaths averted, cases averted and treatment costs averted for each disease in our analysis.

YLL per death


(1) $$YLL=\frac{\sum_{a=1}^{n}(D_{a}\times L_{a})}{D}$$

Where YLL is years of life lost per death, D is annual number of deaths, L is life expectancy, a is age group and n is number of age groups.

YLD per non-treated case


(2) $$YLD=\frac{\sum_{a=1}^{n}\left(I_{a}\times NT_{a}\times DW\right)}{I}$$

Where YLD is years of life lost to disability, I is annual incidence, T is disease duration without treatment, DW is disability weight without treatment, a is age group, and n is number of age groups.

YLD per treated case


(3) $$YLD=\frac{\sum_{a=1}^{n}\left(I_{a}\times T_{a}\times DW\right)}{I}$$

Where YLD is years of life lost to disability, I is annual incidence, T is disease duration with treatment, DW is disability weight with treatment, a is age group, and n is number of age groups.

Cases averted by new vaccines


(4) $$N=\sum_{x=1}^{n}\left({IB}_{x}-{IV}_{x}\right)$$

Where N is cases averted, IB is base case incidence of disease, IV is incidence of disease with a new vaccine, x is year, and n is number of years.

Deaths averted by new vaccines, therapeutics and diagnostics


(5) $$N=\begin{array}{l} \sum_{x=1}^{n}[((IB_{x}\times (1-CB_{x})\times CFR)+(IB_{x}\times CB_{x}\times CFRT))\\ -((IV_{x}\times (1-CD_{x})\times CFR)+(IV_{x}\times CD_{x}\times CFRT))] \end{array}$$

Where N is deaths averted, IB is base case incidence of disease, IV is incidence of disease with a new vaccine, CB is base case treatment coverage of disease, CD is treatment coverage of disease with a new therapeutic and/or diagnostic, CFR is case fatality rate of disease without treatment, CFRT is case fatality rate of disease with treatment, x is year and n is number of years.

YLL averted by new vaccines, therapeutics and diagnostics


(6) $$N=\sum_{x=1}^{n}\left(DB_{x}\times YLL-DT_{x}\times YLL\right)$$

Where N is years of life lost to death averted, DB is base case number of deaths, DT is number of deaths with a new vaccine, therapeutic, and or diagnostic, YLL is years of life lost per death, x is year, and n is number of years.

YLD averted by new vaccines, therapeutics and diagnostics


(7) $$N=\begin{array}{l} \sum_{x=1}^{n}[((IB_{x}\times (1-CB_{x})\times YLD)+(IB_{x}\times CB_{x}\times YLDT))\\ -((IV_{x}\times (1-CD_{x})\times YLD)+(IV_{x}\times CD_{x}\times YLDT))] \end{array}$$

Where N is years of life lost to disability averted, IB is base case incidence of disease, IV is incidence of disease with a new vaccine, CB is base case treatment coverage, CD is treatment coverage with a new therapeutic and or diagnostic, YLD is years of life lost to disability per non-treated case, YLDT is years of life lost to disability per treated case, x is years and n is number of years.

DALYs averted by new vaccines, therapeutics and diagnostics


(8) $$N=\sum_{x=1}^{n}\left({YLL}_{x}+{YLD}_{x}\right)$$

Where N is DALYs averted, YLL is years of life lost to death averted, YLD is years of life lost to disability averted, x is years and n is number of years.

Treatment costs averted


(9) $$C=\begin{array}{l} \sum_{x=1}^{n}[(N_{x}\times K)\\ -(IV_{x}\times CD_{x}-IB_{x}\times CB_{x})\times K] \end{array}$$

Where C is treatment costs averted, N is cases averted by new vaccine, K is cost of treatment per case, IV is incidence of disease with new vaccine, IB is base case incidence of disease, CD is treatment coverage with new therapeutic and or diagnostic, CB is base case treatment coverage, x is year, n is number of years.

### Operational costs, health system strengthening and vaccine procurement costs

We assumed an operational cost would be incurred for each year that the pooled-funding mechanism is in operation. This recurring annual operational cost amounted to US$25 million for design option 1, US$40 million for design option 2 and US$60 million for design option 3. For each design option we also applied a one-time start-up cost amounting to 45% of the annual operational cost. In addition, we assumed an annual investment in health system strengthening would be incurred for the first 5 years in design options 2 and 3. This recurring annual health system strengthening investment amounted to US$100 million for design option 2 and US$250 million for design option 3. All values used for operational costs, start-up costs and health system strengthening investments were informed by KIIs with stakeholders from governments, regulatory bodies, multilateral and global organisations, private pharmaceutical industries, and academic institutions.^11^ Lastly, we assumed a vaccine efficacy of 75% and a vaccine procurement cost of US$10 per dose. Equation 10 shows how we calculated vaccine procurement costs.

Vaccine procurement costs


(10) $$C=\sum_{x=1}^{n}\left(\frac{N_{x}}{E}\right)\times K$$

Where C is vaccine procurement costs, N is cases averted by new vaccine, E is vaccine efficacy, K is vaccine procurement cost per dose, x is year and n is number of years.

### Cost-effectiveness and cost–benefit analysis

**Figure 1:**
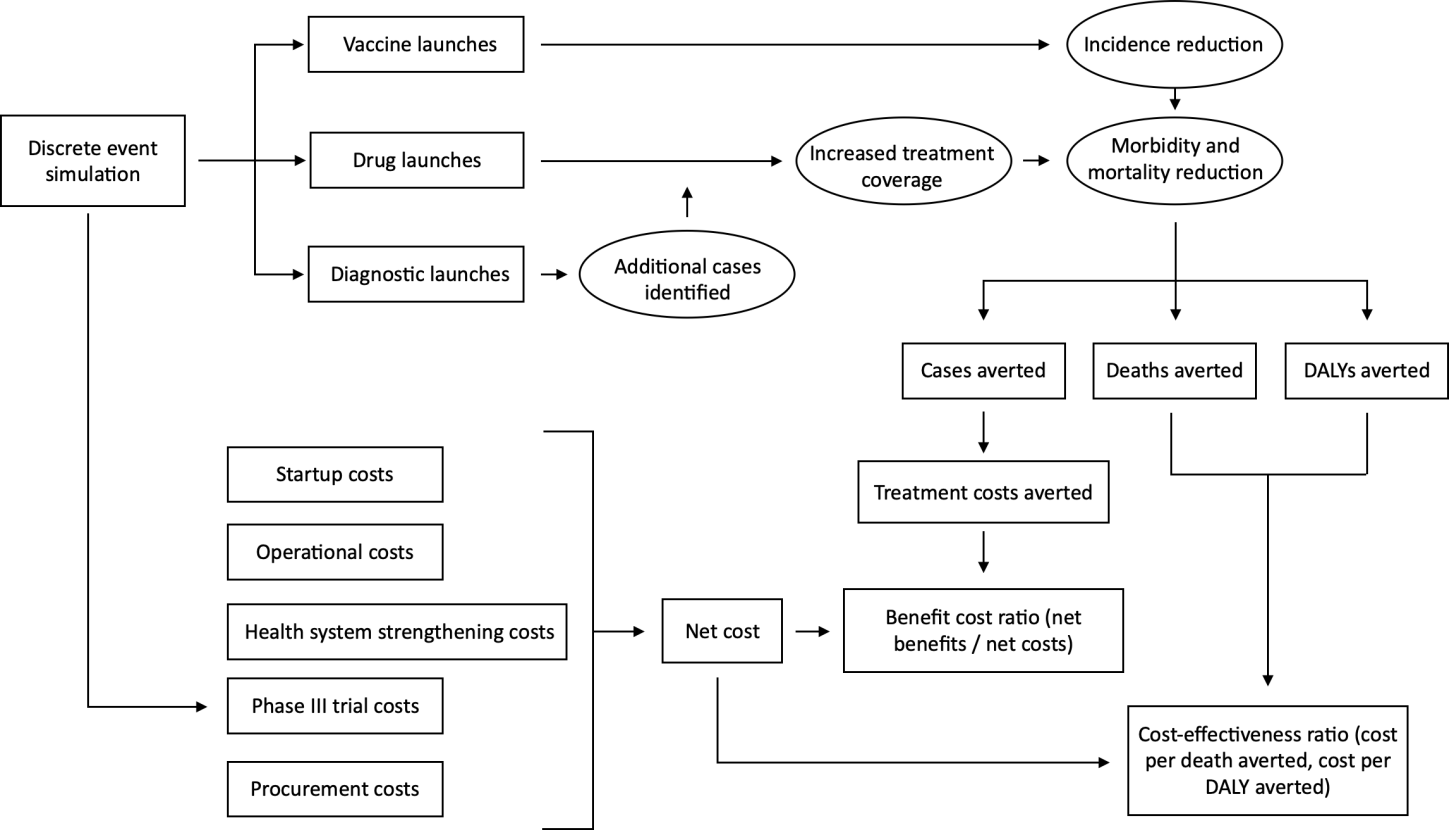
Conceptual diagram of modelling approach. The overall model consists of three main components: (1) the DES model that projects the number of product launches and the clinical trial costs, (2) the economic and health benefits model which projects the potential economic and health benefits from product launches and (3) the economic analyses that calculate the BCRs and ICERs of the different design options. BCRs benefit–cost ratio; DES, discrete event simulation; ICERs, incremental cost-effectiveness ratio.

For each pooled-funding mechanism design option, we calculated the cost per DALY averted and the cost per death averted as well as the incremental cost-effectiveness ratio (ICER) defined as the difference in costs between two design options divided by the difference in effectiveness between two design options.^18^ We also calculated benefit–cost ratios (BCRs) for each design option, defined as the total monetary benefits divided by the total monetary costs. For this analysis, the only monetary benefits we considered were treatment costs averted. All costs, economic benefits and health benefits were discounted at a 3% annual discount rate. All costs are reported in 2020 USD. Figure 1 illustrates our overall approach to the cost-effectiveness and cost–benefit analysis.

### Sensitivity analysis

To account for uncertainty in our model parameters, we conducted a multiway sensitivity analysis with our DES model. Specifically, for each design option, we increased base case phase III clinical trial costs as well as base case preclinical, phase I, phase II and phase III clinical trial durations by 100% for all entity types. This sensitivity analysis allowed us to account for potential delays that prolong clinical trial phases and therefore increase overall costs associated with conducting clinical trials. For each sensitivity analysis, we ran 100 Monte Carlo simulations and averaged product launches across each of the 100 simulations to obtain mean launches per entity type per year.

### Efficiency analysis

We assumed a pooled-funding mechanism could result in clinical trial efficiency gains. Based on a desk review and consultations with product development experts, we established that a pooled-funding mechanism could result in operational and technical efficiencies.^11^ Technical efficiencies could arise if the pooled-funding mechanism funded clinical trials with adaptive designs such as seamless phase II/III trials or adaptive randomisation.^19^ Consultations with R&D stakeholders conducted by our team suggested that adaptive clinical trials can shorten phase durations by 6 months and reduce overall clinical trial costs by 15%. Furthermore, the rapid development of COVID-19 vaccines suggests that phase durations could be significantly reduced when using adaptive designs. Therefore, we modelled two efficiency scenarios: (1) 50% of trials funded by the pooled-funding mechanism use adaptive designs resulting in a 3-month reduction in preclinical, phase I, phase II and phase III clinical trial durations and a 7.5% reduction in phase III trial costs and (2) 100% of trials funded by the pooled-funding mechanism use adaptive designs resulting in a 6-month reduction in preclinical, phases I, II and III clinical trial durations and a 15% reduction in phase III trial costs. Again, for each efficiency analysis, we ran 100 Monte Carlo simulations and averaged product launches across each of the 100 simulations to obtain mean launches per entity type per year. All methods and results from this study are reported in accordance with the Consolidated Health Economic Evaluation Reporting Standards checklist, which can be found in online supplemental appendix 1.

## Results

### Phase III trial costs and expected product launches for each design option

For design option 1, we simulated a total of 116 early stage (preclinical, phase 1 and phase 2) vaccine candidates for HIV, TB, malaria and pneumococcal pneumonia. Twenty-three (20%) candidates were in an advanced preclinical stage, 63 candidates (54%) were in phase I and 30 candidates (26%) were in phase II. The total phase III costs were US$2.8 billion over 11 years. Option 1 resulted in 10 vaccine launches between 2023 and 2029 (table 3). Our DES model predicted the launch of a new pneumococcal vaccine in 2023, followed by an HIV, TB and malaria vaccine in 2026.

**Table 3:** Product candidate portfolio, launches, costs, economic benefits and BCR for each design option, from a societal perspective

	Option 1	Option 2	Option 3
No of early-stage product candidates*	116	327	506
Expected no of product candidates that reach phase III trials†	16	113	172
Expected no of product candidates that launch‡	10	99	147
Phase III trial costs (2020 USD billions)	US$2.80	US$8.96	US$16.83
Net costs (2020 USD billions)	US$43.07	US$50.81	US$117.64
Net benefits (2020 USD billions)	US$238.40	US$197.40	US$296.67
BCR	5.53	3.88	2.52

* Number of product candidates in early stages of development (preclinical, phase I and phase II trials) as of 31 August 2019.

† Based on the actual pipeline without accounting for replenishment. Successful candidates are expected to reach phase III trials between 2022 and 2029.

‡ Expected launches occur between 2023 and 2031.

BCR, benefit–cost ratio.

For design option 2, we simulated 327 early-stage vaccine, therapeutic and diagnostic candidates. Of these, 272 (83%) products were for HIV, TB and malaria, 16 (5%) were for visceral leishmaniasis, 14 (4%) were for Chagas disease, 9 (3%) were for pneumococcal pneumonia, 9 (3%) were for schistosomiasis, 5 (2%) were for dengue and 2 (1%) for leprosy. The total phase III costs were US$8.96 billion. Option 2 resulted in 155 product launches (table 3).

For design option 3, we simulated the entire early-stage product portfolio for PRNDs (506 product candidates). In summary, the highest number of candidates were for malaria (19%), TB (17%), HIV (17%) and Ebola (14%). With the exception of Chagas disease and HIV, all NTDs had less than 10 early-stage candidates and when combined they represented only 11.5% of the early-stage portfolio. The total phase III costs for option 3 were US$16.83 billion. Option 3 resulted in 256 product launches (table 3). Disaggregation of product launches by disease and archetype for each design option are available in online supplemental appendix 1.

### Economic benefits, health benefits, cost-effectiveness ratios and BCRs

We estimated that over 10 years, design option 1 would avert 18.4 million deaths and 516 million DALYs, design option 2 would avert 22.9 million deaths and 674 million DALYs, and design option 3 would avert 26.9 million deaths and 1.03 billion DALYs (table 4). Disaggregation of DALYs averted by disease for each option are available in online supplemental appendix 1.

**Table 4:** Sensitivity and efficiency analysis results for each design option, from a societal perspective

Option 1				
	100% increase in cost and cycle length	Main analysis	50% adaptive trials	100% adaptive trials
Net cost (2020 USD billions)	US$14.71	US$43.07	US$45.28	US$50.06
DALYs averted (millions)	102	516	566	617
Cost per DALY averted (USD 2020)	US$144	US$84	US$80	US$81
Deaths averted (millions)	4.2	18.4	19.8	21.6
Cost per death averted (USD 2020)	US$3491	US$2341	US$2282	US$2312
BCR	3.68	5.53	5.65	5.65

Option 2				
	100% increase in cost and cycle length	Main analysis	50% adaptive trials	100% adaptive trials
Net cost (2020 USD billions)	US$26.60	US$50.81	US$52.89	US$57.60
DALYs averted (millions)	214	674	738	785
Cost per DALY averted (USD 2020)	US$134	US$75	US$72	US$73
Deaths averted (millions)	7.5	22.9	24.7	26.3
Cost per death averted (USD 2020)	US$3808	US$2217	US$2145	US$2190
BCR	0.52	3.88	4.06	4.18

Option 3				
	100% increase in cost and cycle length	Main analysis	50% adaptive trials	100% adaptive trials
Net cost (2020 USD billions)	US$60.10	US$117.64	US$122.00	US$137.79
DALYs averted (millions)	295	1033	1157	1293
Cost per DALY averted (USD 2020)	US$204	US$114	US$105	US$107
Deaths averted (millions)	8.2	26.9	29.0	32.6
Cost per death averted (USD 2020)	US$7315	US$4371	US$4209	US$4233
BCR	0.38	2.52	2.73	2.89

BCR, benefit–cost ratio; DALY, disability-adjusted life-year.

From a societal perspective, design option 1 had a cost per death averted of US$2341 and a cost per DALY averted of US$84. Design option 2 had a cost per death averted of US$2217 and a cost per DALY averted of US$75. Design option 3 had a cost per death averted of US$4371 and a cost per DALY averted of US$114 (table 4). The ICER of option 2 compared with option 1 was US$48.91 and for option 3 compared with option 2 was US$186.00 (online supplemental appendix 1).

From an altruistic investor perspective, option 1 had a cost per death averted of US$152 and a cost per DALY averted of US$5.43. Option 2 had a cost per death averted of US$429 and a cost per DALY averted of US$9.84. Option 3 had a cost per death averted of US$692 and a cost per DALY averted of US$18.61 (online supplemental appendix 1).

Option 1 was projected to avert US$238.40 billion in treatment costs with a net cost of US$43.07. The BCR of option 1 was 5.53. Option 2 was projected to avert US$197.40 billion in treatment costs and had a net cost of US$50.81 billion and a BCR of 3.88. Design option 3 was projected to avert US$296.67 billion in treatment costs and had a net cost of US$117.64 billion and a BCR of 2.52 (table 3).

### Sensitivity analysis

With a 100% increase in phase III trial costs and clinical trial phase durations, option 1 has a net cost of US$14.71 billion and would avert 102 million DALYs between 2021 and 2035 and 4.2 million deaths. The cost per DALY averted increased to US$144, the cost per death averted increased to US$3491 and the BCR decreased to 3.68. Option 2 has a net cost of US$26.60 billion and would avert 214 million DALYs and 7.5 million deaths. The cost per DALY averted and the cost per death averted for option 2 increased to US$134 and US$3808, respectively. The BCR for option 2 decreased to 0.52. Option 3 has a net cost of US$60.10 billion and would avert 295 million DALYs and 8.2 million deaths. The option 3 cost per DALY averted and cost per death averted increased to US$204 and US$7315, respectively, and the BCR decreased to 0.38 (table 4). Ultimately, increased phase III costs and trial durations reduce net costs by 65.8% in option 1, 47.6% in option 2 and 48.9% in option 3, but also reduce DALYs averted by 80.2% in option 1, 68.2% in option 2 and 71.4% in option 3.

### Efficiency analysis

Results from the efficiency analysis are shown in table 4. With 50% adaptive trials, option 1 averts 566 million DALYs and 19.8 million deaths with a net cost of US$45.28 billion. Option 2 averts 738 million DALYs and 24.7 million deaths with a net cost of US$52.89 billion. Option 3 averts 1157 million DALYs and 29.0 million deaths with a net cost of US$122.00 billion. With 100% adaptive trials, option 1 averts 617 million DALYs and 21.6 million deaths with a net cost of US$50.06 billion. Option 2 averts 785 million DALYs and 26.3 million deaths with a net cost of US$57.60 billion. Lastly, with 100% adaptive trials, option 3 averts 1293 million DALYs and 32.6 million deaths with a net cost of US$137.79 billion.

## Discussion

Our study provides evidence on the economic and health benefits of investing in a pooled-funding mechanism to support clinical trials for PRND vaccines, therapeutics and diagnostics. Our results show that investments in PRND clinical trials with concurrent investments in health system strengthening would have a significant impact on global health. Investing in vaccine late-stage clinical trials for HIV, TB, malaria and pneumococcal pneumonias could avert 18.4 million deaths and 516 million DALYs globally over the period 2019–2030. Investing in vaccine and therapeutic late-stage clinical trials for HIV, TB, malaria, pneumococcal pneumonias and NTDs could avert 22.9 million deaths and 674 million DALYs globally. Lastly, expanding the investments to include vaccine, therapeutic and diagnostic late-stage clinical trials for 30 PRNDs could avert 26.9 million deaths and 1.03 billion DALYs globally. Not only does each investment scenario produce substantial global health benefits, but each is also cost-effective.

The investments modelled in each pooled-funding mechanism design option are cost-effective in comparison to both established international cost-effectiveness thresholds and other widely implemented global health interventions. The WHO, for example, defines an intervention as cost-effective if the cost-effectiveness ratio (cost per DALY averted) is less than three times the implementing country’s gross domestic product (GDP) per capita.^20^ With a societal perspective, the cost per DALY averted ranges from US$84 for the most restrictive design option (option 1) to US$114 for the most inclusive design option (option 3). Thus, the cost-effectiveness ratios for a pooled-funding mechanism for PRNDs remain well below the GDP per capita of most countries worldwide.^21^ In addition, these ratios are similar in magnitude to other widely implemented global health interventions including bed nets for malaria and antiretrovirals for HIV which have costs per DALY averted of US$6.48–US$22.04 and US$453.74–US$648.20, respectively.^22^

We provide a conservative estimate of both the economic and health benefits of late-stage clinical trial investments. While our study models the launch of PRND products from the R&D pipeline, our analysis does not account for the effects of multiple, similar products entering the marketplace at the same time. Vaccines, therapeutics, or diagnostics with similar target product profiles, and which enter the marketplace at the same time, tend to drive down costs.^23 24^ Lower product costs may also increase access to treatment. Hence, the late-stage clinical trial investments modelled in this study would likely be more cost-effective and the health benefits resulting from these investments would likely be higher. There are also additional economic benefits unaccounted for in this study. The societal perspective benefits modelled in our study only include treatment costs averted through reduced disease incidence resulting from new PRND products that enter the market. In reality, a reduced disease incidence would also spur economic productivity through reduced illness-related work absenteeism. Thus, the societal perspective benefits presented in this study are likely an underestimate.

Our results also show that the health benefits gained through investments in late-stage clinical trials for PRNDs are even greater if adaptive trial designs are used. The efficiency gains resulting from adaptive trial designs include reductions in overall costs and phase length times. These gains create a shorter timeline between product entry into the R&D pipeline and product launch, thus allowing public health benefits to accrue sooner. Our study shows that by using only adaptive clinical trials option 1 can avert an additional 101 million DALYs and 3.2 million deaths, option 2 can avert an additional 111 million DALYs and 3.4 million deaths, and option 3 can avert an additional 260 million DALYs and 5.7 million deaths. Although our modelled efficiency gains were based on empirical evidence from consultations and peer-reviewed literature, actual efficiency gains are likely to be more substantial. The COVID-19 pandemic has shown that parallel clinical trials, harmonised trial protocols across product candidates, targeted trial site selection and streamlined regulatory reviews can significantly reduce clinical trial time.^25^ These efficiencies resulted in the first COVID-19 vaccine being administered to the public less than 1 year after sequencing of the SARS-CoV-2 genome.^26^ With sufficient demand and political will, similar efficiencies could expedite PRND product development and generate PRND related health benefits sooner.

The COVID-19 pandemic has also opened a window of opportunity for launching a global pooled-funding mechanism to fund PRND product development. While the pandemic may divert funds away from PRND product development in the short term, it has sparked action at the international level to redefine global R&D governance in a way that could prove beneficial to PRNDs in the long term. For example, the African Union and Africa CDC, in response to the pandemic, created the Partnership for African Vaccine Manufacturing (PAVM) not only to increase manufacturing capacity on the African continent, but also to establish research and academic hubs that will enable Africa to develop its own vaccine products.^27^ The African Union has also planned the establishment of an African Medicines Agency (AMA) to improve and harmonise pharmaceutical regulatory capacity across African countries.^28^ In addition, the WHO is seeking to establish technology transfer hubs in LMICs to facilitate vaccine production in those countries.^29^ For example, the WHO and its partners have established a technology transfer mRNA vaccine hub in Cape Town, South Africa, which aims to ‘build capacity in LMICs to produce mRNA vaccines through a centre of excellence and training’.^30^ Ultimately, embedding conversations around PRND products and their cost-effectiveness into new initiatives like PAVM, AMA and those being led by the WHO could generate demand for and could help establish a new system for PRND funding.

To the best of our knowledge, this is the first study to estimate the economic and health benefits of a pooled-funding mechanism to support late-stage clinical trials of PRND products. Consequently, the results of this study could help inform future decision-making on health-related investments. Nevertheless, there are important limitations to consider when interpreting our results. First, our analysis is based on the 2019 portfolio of PRND product candidates, and the economic and health benefits are only projected over a 10-year period. While a shorter or longer time horizon would accrue less or more benefits, respectively, the actual benefits accrued would also depend on the portfolio of PRND candidates in the R&D pipeline at the time of the establishment of the pooled-funding mechanism. Second, we assumed that early stages of the R&D pipeline would be replenished at a rate of ten new PRND products per year for the first 5 years of the time horizon. This assumption may not hold true in reality, and the actual rate of replenishment may be less or more. Our analysis, however, only accounts for the first launch of each unique product archetype and therefore limits the overestimation of benefits that may result from a rate of pipeline replenishment that is too high. Third, the investment options modelled in this study are not necessarily the most cost-effective or efficient strategies for reducing the global burden of PRNDs. Any investment in PRNDs may be more cost-effective than the status quo, and additional analyses would be required to compare the cost-effectiveness of other investment strategies to those modelled in this study. Lastly, while not necessarily a limitation, it is important to reiterate that the economic returns modelled in this study are returns to humanity and not returns to any investor or investment mechanism. The pooled-funding mechanism evaluated in this study functions as a nonprofit, prioritising health and economic returns to humanity over personal returns to investors.

## Conclusion

Overall, our study supports the implementation of a global pooled-funding mechanism for late-stage clinical trials of PRND vaccines, therapeutics and diagnostics as an alternative to current PRND funding models. The burden of PRND-related morbidity and mortality in LMICs is substantial, and current funding is insufficient to move needed PRND product candidates through the R&D pipeline. Improved coordination generated by a global pooled-funding mechanism can streamline PRND financing thereby bringing new PRND products to market that can avert millions of deaths, billions of DALYs and billions in treatment costs worldwide.

## Supplementary material

10.1136/bmjgh-2023-011842 online supplemental appendix 1

## Data Availability

Data are available on reasonable request.
