# Supplementary material for: Investing in a global pooled-funding mechanism for late-stage clinical trials of poverty-related and neglected diseases: an economic evaluation
Source: BMJ Glob Health. 2023 May 29;8(5):e011842. doi: 10.1136/bmjgh-2023-011842 (PMC10230859; doi:10.1136/bmjgh-2023-011842)
Supplement: online supplemental appendix 1 [file bmjgh-2023-011842supp001.pdf]

Appendix

CHEERS Checklist

Topic	No.	Item	Location where item is reported
**Title**			
	1	Identify the study as an economic evaluation and specify the interventions being compared.	Page 1
**Abstract**			
	2	Provide a structured summary that highlights context, key methods, results, and alternative analyses.	Page 2
**Introduction**			
**Background and objectives**	3	Give the context for the study, the study question, and its practical relevance for decision making in policy or practice.	Page 3
**Methods**			
**Health economic analysis plan**	4	Indicate whether a health economic analysis plan was developed and where available.	Page 4
**Study population**	5	Describe characteristics of the study population (such as age range, demographics, socioeconomic, or clinical characteristics).	Page 4-5
**Setting and location**	6	Provide relevant contextual information that may influence findings.	Page 4-5
**Comparators**	7	Describe the interventions or strategies being compared and why chosen.	Page 4, Table 1
**Perspective**	8	State the perspective(s) adopted by the study and why chosen.	Page 5, Table 2
**Time horizon**	9	State the time horizon for the study and why appropriate.	Page 6
**Discount rate**	10	Report the discount rate(s) and reason chosen.	Page 10
**Selection of outcomes**	11	Describe what outcomes were used as the measure(s) of benefit(s) and harm(s).	Pages 5
**Measurement of outcomes**	12	Describe how outcomes used to capture benefit(s) and harm(s) were measured.	Pages 7-10
**Valuation of outcomes**	13	Describe the population and methods used to measure and value outcomes.	Pages 7-10

Topic	No.	Item	Location where item is reported
**Measurement and valuation of resources and costs**	14	Describe how costs were valued.	Pages 6-10
**Currency, price date, and conversion**	15	Report the dates of the estimated resource quantities and unit costs, plus the currency and year of conversion.	Page 10
**Rationale and description of model**	16	If modelling is used, describe in detail and why used. Report if the model is publicly available and where it can be accessed.	Pages 6
**Analytics and assumptions**	17	Describe any methods for analysing or statistically transforming data, any extrapolation methods, and approaches for validating any model used.	Pages 6-10
**Characterising heterogeneity**	18	Describe any methods used for estimating how the results of the study vary for subgroups.	NA – necessary data not available to determine variability of effects from product launches across subgroups
**Characterising distributional effects**	19	Describe how impacts are distributed across different individuals or adjustments made to reflect priority populations.	NA – necessary data not available to determine varying impacts of product launches across individuals
**Characterising uncertainty**	20	Describe methods to characterise any sources of uncertainty in the analysis.	Page 10
**Approach to engagement with patients and others affected by the study**	21	Describe any approaches to engage patients or service recipients, the general public, communities, or stakeholders (such as clinicians or payers) in the design of the study.	Pages 6, 9
**Results**			
**Study parameters**	22	Report all analytic inputs (such as values, ranges, references) including uncertainty or distributional assumptions.	Table 3, Appendix
**Summary of main results**	23	Report the mean values for the main categories of costs and outcomes of interest and summarise them in the most appropriate overall measure.	Pages 11-13, Table 4
**Effect of uncertainty**	24	Describe how uncertainty about analytic judgments, inputs, or projections affect findings. Report the effect of choice of discount rate and time horizon, if applicable.	Page 12-13, Table 5

Topic	No.	Item	Location where item is reported
**Effect of engagement with patients and others affected by the study**	25	Report on any difference patient/service recipient, general public, community, or stakeholder involvement made to the approach or findings of the study	Page 4
**Discussion**			
**Study findings, limitations, generalisability, and current knowledge**	26	Report key findings, limitations, ethical or equity considerations not captured, and how these could affect patients, policy, or practice.	Pages 14-15
**Other relevant information**			
**Source of funding**	27	Describe how the study was funded and any role of the funder in the identification, design, conduct, and reporting of the analysis	Page 16
**Conflicts of interest**	28	Report authors conflicts of interest according to journal or International Committee of Medical Journal Editors requirements.	Page 16

From: Husereau D, Drummond M, Augustovski F, et al. Consolidated Health Economic Evaluation Reporting Standards 2022 (CHEERS 2022) Explanation and Elaboration: A Report of the ISPOR CHEERS II Good Practices Task Force. Value Health 2022;25. doi:10.1016/j.jval.2021.10.008

Prevalence, incidence, and annual death inputs for each disease

Disease	Point prevalence	Incidence	Annual number of deaths	Year	Source
HIV/AIDS	36,822,237	1,942,071	954,492	2017	IHME, GBD results tool
Malaria	136,085,123	208,768,201	619,827	2017	IHME, GBD results tool
Tuberculosis	1,929,208,623	8,965,814	1,183,672	2017	IHME, GBD results tool
Pneumonia	n/a	471,825,514	2,558,606	n/a	IHME, GBD results tool
Chagas disease	6,196,959	162,470	7,853	2017	IHME, GBD results tool
Schistosomiasis	142,788,542	71,385,000	8,837	2016	GBD 2016
Leishmaniasis	4,130,197	669,058	7,527	2017	IHME, GBD results tool
Dengue	6,267,410	104,771,911	40,467	2017	IHME, GBD results tool
Leprosy	518,527	48,477	4,000	2017	IHME, GBD results tool Engers H, Morel CM. Leprosy. Nat Rev Microbiol. 2003;1(2):94-95. doi:10.1038/nrmicro764
Shigellosis	NA	269,191,131	212,438	2016	Khalil IA, Troeger C, Blacker BF, et al. Morbidity and mortality due to shigella and enterotoxigenic Escherichia coli diarrhoea: the Global Burden of Disease Study 1990-2016 [published correction appears in Lancet Infect Dis. 2018 Oct 30;:]. Lancet Infect Dis. 2018;18(11):1229-1240. doi:10.1016/S1473-3099(18)30475-4
Ebola	NA	20,200	7,905	2014	Cenciarelli O, Pietropaoli S, Malizia A, et al. Ebola virus disease 2013-2014 outbreak in west Africa: an analysis of the epidemic spread and response. Int J Microbiol. 2015;2015:769121. doi:10.1155/2015/769121
Hepatitis C	135,447,784	6,527,210	580,052	2017	IHME, GBD results tool
Enterotoxigenic E.coli (ETEC)	NA	222,637,561	51,186	2010	Khalil IA, Troeger C, Blacker BF, et al. Morbidity and mortality due to shigella and enterotoxigenic Escherichia coli diarrhoea: the Global Burden of Disease Study 1990-2016 [published correction appears in Lancet Infect Dis. 2018 Oct 30;:]. Lancet

					Infect Dis. 2018;18(11):1229-1240. doi:10.1016/S1473-3099(18)30475-4
Non-typhoidal Salmonella (NTS)	NA	534,595	120,281	2017	IHME, GBD results tool
HAT	4,896	3,322	1,364	2017	IHME, GBD results tool
Onchocerciasis	20,938,147	1,017,375	60,025	2017	IHME, GBD results tool
Cholera	NA	2,800,000	75,772	2017	IHME, GBD results tool
Hookworm	229,217,130	86,972,676	65,000	2016	Bartsch SM, Hotez PJ, Asti L, et al. The Global Economic and Health Burden of Human Hookworm Infection. PLoS Negl Trop Dis. 2016;10(9):e0004922. Published 2016 Sep 8. doi:10.1371/journal.pntd.0004922 Stanley Plotkin, David J. Diemert, Jeffrey M. Bethony, Peter J. Hotez, Hookworm Vaccines, Clinical Infectious Diseases, Volume 46, Issue 2, 15 January 2008, Pages 282–288, https://doi.org/10.1086/524070
Meningitis	10,572,886	5,045,411	288,021	2017	IHME, GBD results tool
Rheumatic fever	39,345,369	1,311,253	285,517	2017	IHME, GBD results tool
Multiple diarrhoeal diseases	93,472,768	6,292,936,672	1,569,556	2017	IHME, GBD results tool
Buruli Ulcer	NA	2,708	14	2017	Global Health Observatory Data Repository
Trachoma	3,818,880	2,034,879	50,870	2017	Gouda H, Powles J, Barendregt J, Emerson P, Ngondi J. The burden of trachoma in South Sudan: assessing the health losses from a condition of graded severity. PLoS Negl Trop Dis. 2012;6(3):e1538. doi:10.1371/journal.pntd.0001538 WHO Alliance for the Global Elimination of Trachoma by 2020: progress report on elimination of trachoma, 2014–2016

Typhoid & paratyphoid	387,451	14,321,147	135,922	2017	IHME, GBD results tool
Cryptosporidiosis	NA	64,003,709	27,553	2010	Kirk MD, Pires SM, Black RE, et al. World Health Organization Estimates of the Global and Regional Disease Burden of 22 Foodborne Bacterial, Protozoal, and Viral Diseases, 2010: A Data Synthesis [published correction appears in PLoS Med. 2015 Dec;12(12):e1001940]. PLoS Med. 2015;12(12):e1001921. Published 2015 Dec 3. doi:10.1371/journal.pmed.1001921
Multiple salmonella infections	NA	25,811,160	178,215	2010	Kirk MD, Pires SM, Black RE, et al. World Health Organization Estimates of the Global and Regional Disease Burden of 22 Foodborne Bacterial, Protozoal, and Viral Diseases, 2010: A Data Synthesis [published correction appears in PLoS Med. 2015 Dec;12(12):e1001940]. PLoS Med. 2015;12(12):e1001921. Published 2015 Dec 3. doi:10.1371/journal.pmed.1001921
Hepatitis B	448,571,213	147,666,509	799,009	2017	IHME, GBD results tool
Herpes Simplex-2	955,894,784.19	77,696,683.76		2017	IHME, GBD results tool
Gonorrhea	47,269,180.69	137,221,507.51	3,019.11	2017	IHME, GBD results tool
Chlamydia	109,822,037.14	297,131,257.70	1,050.55	2017	IHME, GBD results tool

Disability weight inputs for each disease.

Disease	Health state	Disability weight	Reference
HIV	Acute HIV (entry)	0.012	IHME
	CD4 > 500	0.078	IHME
	CD4 500-350	0.274	IHME
	CD4 350-200	0.582	IHME
	CD4 <200	0.582	IHME
Tuberculosis	Drug-susceptible tuberculosis	0.333	IHME
	Multidrug-resistant tuberculosis without extensive drug resistance	0.333	IHME
	Extensively drug-resistant tuberculosis	0.333	IHME
Malaria	Mild malaria	0.006	IHME
	Moderate malaria	0.051	IHME
	Severe malaria	0.133	IHME
	Severe motor impairment due to malaria	0.402 (0.268-0.545)	IHME
Pneumonia	Moderate lower respiratory infections	0.051 (0.032-0.074)	IHME
	Severe lower respiratory infections	0.133 (0.088-0.190)	IHME
Chagas disease	Asymptomatic Chagas disease	n/a	IHME
	Acute Chagas disease	0.051 (0.032-0.074)	IHME
	Moderate chronic digestive disease due to Chagas disease	0.114 (0.078-0.159)	IHME
	Moderate heart failure due to Chagas disease	0.072 (0.047-0.103)	IHME
	Severe heart failure due to Chagas disease	0.179 (0.122-0.251)	IHME
	Treated heart failure due to Chagas disease	0.049 (0.031-0.072)	IHME
	Mild heart failure due to Chagas disease	0.041 (0.026-0.062)	IHME
Schistosomiasis	Severe anemia due to schistosomiasis	0.149 (0.101-0.209)	IHME
	Moderate anemia due to schistosomiasis	0.052 (0.034-0.076)	IHME
	Bladder pathology due to schistosomiasis	0.011 (0.005-0.021)	IHME
	Hydronephrosis due to schistosomiasis	0.011 (0.005-0.021)	IHME
	Hepatomegaly due to schistosomiasis	0.011 (0.005-0.021)	IHME
	Hematemesis due to schistosomiasis	0.325 (0.209-0.462)	IHME
	Ascites due to schistosomiasis	0.114 (0.078-0.159)	IHME
	Mild diarrhea due to schistosomiasis	0.074 (0.049-0.104)	IHME
	Mild schistosomiasis	0.006 (0.002-0.012)	IHME
	Mild anemia due to schistosomiasis	0.004 (0.001-0.008)	IHME
Leishmaniasis	Severe visceral leishmaniasis	0.133 (0.088-0.190)	IHME
	Moderate visceral leishmaniasis	0.051 (0.032-0.074)	IHME
	Cutaneous and mucocutaneous leishmaniasis	0.067 (0.044-0.096)	IHME
Dengue	Moderate dengue	0.051 (0.032-0.074)	IHME
	Severe dengue	0.133 (0.088-0.190)	IHME
Leprosy	Disfigurement level 1 due to leprosy	0.011 (0.005-0.021)	IHME
	Disfigurement level 2 due to leprosy	0.067 (0.044-0.096)	IHME
Buruli Ulcer	Mild decubitus ulcer	0.027 (0.015-0.042)	IHME
	Moderate decubitus ulcer	0.188 (0.125-0.267)	

	Severe decubitus ulcer	0.576 (0.401-0.731)	
Cholera	Mild diarrheal diseases	0.074 (0.049-0.104)	IHME
	Severe diarrheal diseases	0.247 (0.164-0.348)	IHME
	Moderate diarrheal diseases	0.188 (0.125-0.264)	IHME
Cryptosporadiasis	Mild diarrheal diseases	0.074 (0.049-0.104)	IHME
	Severe diarrheal diseases	0.247 (0.164-0.348)	IHME
	Moderate diarrheal diseases	0.188 (0.125-0.264)	IHME
Ebola	Ebola cases	0.133 (0.088-0.190)	IHME
Enterotoxigenic E. coli	Mild diarrheal diseases	0.074 (0.049-0.104)	IHME
	Severe diarrheal diseases	0.247 (0.164-0.348)	IHME
	Moderate diarrheal diseases	0.188 (0.125-0.264)	IHME
Sleeping Sickness (HAT)	Sleeping sickness due to Trypanosoma brucei rhodesiense	0.542 (0.374-0.702)	IHME
	Skin disfigurement due to Trypanosoma brucei rhodesiense	0.027 (0.015-0.042)	IHME
	Sleeping sickness due to Trypanosoma brucei gambiense	0.542 (0.374-0.702)	IHME
	Skin disfigurement due to Trypanosoma brucei gambiense	0.027 (0.015-0.042)	IHME
Hepatitis C	Severe acute hepatitis C	0.133 (0.088-0.190)	IHME
	Moderate acute hepatitis C	0.051 (0.032-0.074)	IHME
	Terminal phase of liver cancer due to hepatitis C	0.540 (0.377-0.687)	IHME
	Controlled phase of liver cancer due to hepatitis C	0.049 (0.031-0.072)	IHME
	Cirrhosis and other chronic liver diseases due to hepatitis C, decompensated	0.178 (0.123-0.250)	IHME
Hookworm	Heavy infestation of hookworm	0.027 (0.015-0.043)	IHME
	Severe wasting due to hookworm disease	0.128 (0.082-0.183)	IHME
	Moderate anemia due to hookworm disease	0.052 (0.034-0.076)	IHME
	Severe anemia due to hookworm disease	0.149 (0.101-0.209)	IHME
Meningitis			IHME
Multiple diarrhoeal diseases	Mild diarrheal diseases	0.074 (0.049-0.104)	IHME
	Severe diarrheal diseases	0.247 (0.164-0.348)	IHME

	Moderate diarrheal diseases	0.188 (0.125-0.264)	IHME
Multiple salmonella infections	Mild diarrheal diseases	0.074 (0.049-0.104)	IHME
	Severe diarrheal diseases	0.247 (0.164-0.348)	IHME
	Moderate diarrheal diseases	0.188 (0.125-0.264)	IHME
Non-typhoidal salmonella	Mild diarrheal diseases	0.074 (0.049-0.104)	IHME
	Severe diarrheal diseases	0.247 (0.164-0.348)	IHME
	Moderate diarrheal diseases	0.188 (0.125-0.264)	IHME
Onchocerciasis	Severe vision impairment due to onchocerciasis	0.184 (0.125-0.258)	IHME
	Blindness due to onchocerciasis	0.187 (0.124-0.260)	IHME
	Mild skin disease without itch due to onchocerciasis	0.011 (0.005-0.021)	IHME
	Moderate skin disease due to onchocerciasis	0.188 (0.125-0.267)	IHME
	Mild skin disease due to onchocerciasis	0.027 (0.015-0.042)	IHME
	Moderate vision impairment due to onchocerciasis	0.031 (0.019-0.049)	IHME
Rheumatic Fever	No sequelae listed	0.685 (0.592-0.769)	Ock M, Lee JY, Oh IH, Park H, Yoon SJ, Jo MW. Disability Weights Measurement for 228 Causes of Disease in the Korean Burden of Disease Study 2012. J Korean Med Sci. 2016;31 Suppl 2(Suppl 2):S129-S138.doi:10.3346/jkms.2016.31.S2.S129"
Shigellosis	Mild diarrheal diseases	0.074 (0.049-0.104)	IHME
	Severe diarrheal diseases	0.247 (0.164-0.348)	IHME
	Moderate diarrheal diseases	0.188 (0.125-0.264)	IHME
Trachoma	Moderate vision impairment due to trachoma	0.031 (0.019-0.049)	IHME
Typhoid and Paratyphoid	Acute typhoid infection	0.051 (0.032-0.074)	IHME
	Severe typhoid fever	0.133 (0.088-0.190)	IHME
	Intestinal perforation due to typhoid	0.324 (0.220-0.442)	IHME
	Gastrointestinal bleeding due to typhoid	0.325 (0.209-0.462)	IHME

Hepatitis B	Severe acute hepatitis B	0.133<br>(0.088-0.190)	IHME
	Terminal phase of liver cancer due to hepatitis B	0.540<br>(0.377-0.687)	IHME
	Controlled phase of liver cancer due to hepatitis B	0.049<br>(0.031-0.072)	IHME
	Cirrhosis and other chronic liver diseases due to hepatitis B, decompensated	0.178<br>(0.123-0.250)	IHME
Herpes Simplex 2	Moderate infection due to initial genital herpes episode	0.051<br>(0.032-0.074)	IHME
	Symptomatic genital herpes	0.006<br>(0.002-0.012)	IHME
Gonnorhea	Moderate pelvic inflammatory diseases due to gonococcal infection	0.114<br>(0.078-0.159)	IHME
	Mild gonococcal infection	0.006<br>(0.002-0.012)	IHME
	Secondary infertility due to gonococcal infection	0.005<br>(0.002-0.011)	IHME
	Primary infertility due to gonococcal infection	0.008<br>(0.003-0.015)	IHME
Chlamydia	Epididymo-orchitis due to chlamydial infection	0.128<br>(0.086-0.180)	IHME
	Moderate pelvic inflammatory diseases due to chlamydial infection	0.114<br>(0.078-0.159)	IHME
	Severe pelvic inflammatory diseases due to chlamydial infection	0.324<br>(0.220-0.442)	IHME
	Mild chlamydial infection	0.006<br>(0.002-0.012)	IHME
	Secondary infertility due to chlamydial infection	0.005<br>(0.002-0.011)	IHME
	Primary infertility due to chlamydial infection	0.008<br>(0.003-0.015)	IHME

Treatment cost inputs for each disease

Disease	Treatment cost (2020 USD)	Notes	Reference
HIV	$336.00	Average ART cost per person year across different disease states (CD4 >500 to <200)	Ross JM, Ying R, Celum CL, et al. Modeling HIV disease progression and transmission at population-level: The potential impact of modifying disease progression in HIV treatment programs. Epidemics. 2018;23:34-41. doi:10.1016/j.epidem.2017.12.001
Tuberculosis	$1,251.00	Weighted average of treatment cost per case for drug sensitive TB and MDR-TB	WHO: Global TB Report 2019
Malaria	$72.00	Weighted average of treatment cost of moderate malaria and severe malaria	White MT, Conteh L, Cibulskis R, Ghani AC. Costs and cost-effectiveness of malaria control interventions--a systematic review. Malar J. 2011;10:337. Published 2011 Nov 3. doi:10.1186/1475-2875-10-337
Pneumonia	$81.00	NA	Anh, Dang Duc, et al. "Treatment costs of pneumonia, meningitis, sepsis, and other diseases among hospitalized children in Viet Nam." Journal of health, population, and nutrition 28.5 (2010): 436.<br><br>Tichopad, Ales, et al. "Clinical and economic burden of community-acquired pneumonia among adults in the Czech Republic, Hungary, Poland and Slovakia." PLoS One 8.8 (2013).
Chagas disease	$286.00	Average cost per person year across different disease states	Wilson LS, Strosberg AM, Barrio K. Cost-effectiveness of Chagas disease interventions in latin america and the Caribbean: Markov models. Am J Trop Med Hyg. 2005;73(5):901-910.
Schistosomiasis	$4.40	Cost per person	Salari P, Fürst T, Knopp S, Utzinger J, Tediosi F. Cost of interventions to control schistosomiasis: A systematic review of the literature. PLoS Negl Trop Dis. 2020;14(3):e0008098. Published 2020 Mar 30. doi:10.1371/journal.pntd.0008098
Leishmaniasis	$150.93	Average cost per person across different treatment strategies	Meheus F, Balasegaram M, Olliaro P, et al. Cost-effectiveness analysis of combination therapies for visceral leishmaniasis in the Indian subcontinent. PLoS Negl Trop Dis. 2010;4(9):e818. Published 2010 Sep 7. doi:10.1371/journal.pntd.0000818
Dengue	$263.00	Average cost per patient for moderate dengue and severe dengue	Lee JS, Mogasale V, Lim JK, et al. A multi-country study of the economic burden of dengue fever: Vietnam, Thailand, and Colombia. PLoS Negl Trop Dis. 2017;11(10):e0006037. Published 2017 Oct 30. doi:10.1371/journal.pntd.0006037
Leprosy	$309.70	Cost of treatment per person per year	Xiong M, Li M, Zheng D, et al. Evaluation of the economic burden of leprosy among migrant and resident patients in Guangdong Province, China. BMC Infect Dis. 2017;17(1):760. Published 2017 Dec 11. doi:10.1186/s12879-017-2869-8
Shigellosis	$1.11	Outpatient treatment cost for diarrheal disease	Baral, Ranju, et al. "Cost of illness for childhood diarrhea in low-and middle-income countries: a systematic review of evidence and modelled estimates." BMC Public Health 20 (2020): 1-13.
Ebola	$915.35	Extensive supportive care EVD treatment and PPE costs per case	Bartsch, Sarah M., Katrin Gorham, and Bruce Y. Lee. "The cost of an Ebola case." Pathogens and global health 109.1 (2015): 4-9.
Hepatitis C	$980.00	12-week course of sofobuvir for countries like Mongolia, Egypt, Pakistan.	Iyengar, Swathi, et al. "Prices, costs, and affordability of new medicines for hepatitis C in 30 countries: an economic analysis." PLoS medicine 13.5 (2016): e1002032.
Enterotoxigenic E. coli	$36.56	Outpatient treatment cost for diarrheal disease	Baral, Ranju, et al. "Cost of illness for childhood diarrhea in low-and middle-income countries: a systematic review of evidence and modelled estimates." BMC Public Health 20 (2020): 1-13.
Non-typhoidal salmonella	$20.91	Cost per treatment per case (USD 2016). We used estimates for typhoidal salmonella	Luthra K, Watts E, Debellut F, Pecenka C, Bar-Zeev N, Constenla D. A Review of the Economic Evidence of Typhoid Fever and Typhoid Vaccines. Clin Infect Dis. 2019;68(Suppl 2):S83-S95. doi:10.1093/cid/ciy1122
Sleeping Sickness (HAT)	$845.99	Average total cost of Elfornithine administration.	Keating, Joseph, et al. "Human African trypanosomiasis prevention, treatment and control costs: a systematic review." Acta tropica 150 (2015): 4-13.
Onchocerciasis	$38.80	One dose of ivermectin	Keating, Joseph, et al. "Lymphatic filariasis and onchocerciasis prevention, treatment, and control costs across diverse settings: a systematic review." Acta tropica 135 (2014): 86-95.

Cholera	$34.04		Ilboudo, Patrick G., et al. "Cost-of-illness of cholera to households and health facilities in rural Malawi." PloS one 12.9 (2017).<br><br>Schaetti, Christian, et al. "Costs of illness due to cholera, costs of immunization and cost-effectiveness of an oral cholera mass vaccination campaign in Zanzibar." PLoS neglected tropical diseases 6.10 (2012).<br><br>Sarker, Abdur Razzaque, et al. "Cost of illness for cholera in a high risk urban area in Bangladesh: an analysis from household perspective." BMC infectious diseases 13.1 (2013): 518.
Hookworm	$0.31	Cost per round of treatment for pre school-age child	Hall A, Horton S, de Silva N (2009) The Costs and Cost-Effectiveness of Mass Treatment for Intestinal Nematode Worm Infections Using Different Treatment Thresholds. PLOS Neglected Tropical Diseases 3(3): e402. https://doi.org/10.1371/journal.pntd.0000402
Meningitis	$1,749.17	Average treatment cost per infection across 144 LMICs (2012 USD)	Portnoy A, Jit M, Lauer J, et al. Estimating costs of care for meningitis infections in low- and middle-income countries. Vaccine. 2015;33 Suppl 1:A240-A247. doi:10.1016/j.vaccine.2014.11.061
Rheumatic Fever	$15,081.73	RHD admission +RHD valve surgery + RHD medical management	Irlam, James, et al. "Primary prevention of acute rheumatic fever and rheumatic heart disease with penicillin in South African children with pharyngitis: a cost-effectiveness analysis." Circulation: Cardiovascular Quality and Outcomes 6.3 (2013): 343-351.
Multiple diarrhoeal diseases	$36.56	Used outpatient cost of illness for diarrheal diseases	Baral, Ranju, et al. "Cost of illness for childhood diarrhea in low-and middle-income countries: a systematic review of evidence and modelled estimates." BMC Public Health 20 (2020): 1-13.
Buruli Ulcer	$4,058.30	Buruli Ulcer (severity NR)	Omansen, Till F., et al. "Global Epidemiology of Buruli Ulcer, 2010–2017, and Analysis of 2014 WHO Programmatic Targets." Emerging infectious diseases 25.12 (2019): 2183.<br><br>Drummond, Christina, and James RG Butler. "Mycobacterium ulcerans treatment costs, Australia." Emerging infectious diseases 10.6 (2004): 1038.<br><br>Asiedu, Kingsley, and Samuel Etuaful. "Socioeconomic implications of Buruli ulcer in Ghana: a three-year review." The American journal of tropical medicine and hygiene 59.6 (1998): 1015-1022.
Trachoma	$35.29	Cost per patient of targeted azythromycin treatment in regions of Africa with high adult mortality and high child mortality.	Baltussen RM, Sylla M, Frick KD, Mariotti SP. Cost-effectiveness of trachoma control in seven world regions. Ophthalmic Epidemiol. 2005;12(2):91-101. doi:10.1080/09286580590932761
Typhoid and Paratyphoid	$20.91	Cost per treatment per case (USD 2016)	Luthra K, Watts E, Debellut F, Pecenka C, Bar-Zeev N, Constenla D. A Review of the Economic Evidence of Typhoid Fever and Typhoid Vaccines. Clin Infect Dis. 2019;68(Suppl 2):S83-S95. doi:10.1093/cid/ciy1122
Cryptosporadiosis	$24.77	Symptomatic cryptospordiosis	Rafferty, Ellen R., et al. "Pediatric cryptosporidiosis: an evaluation of health care and societal costs in Peru, Bangladesh and Kenya." PloS one 12.8 (2017).
Multiple salmonella infections	$20.91	Cost per treatment per case (USD 2016)	Luthra K, Watts E, Debellut F, Pecenka C, Bar-Zeev N, Constenla D. A Review of the Economic Evidence of Typhoid Fever and Typhoid Vaccines. Clin Infect Dis. 2019;68(Suppl 2):S83-S95. doi:10.1093/cid/ciy1122
Hepatitis B	$34.84	Median cost per patient per year of tenofovir treatment in 2016.	https://www.who.int/news-room/fact-sheets/detail/hepatitis-b
Herpes Simplex 2	$11.74	Average cost per treatment across LMICs (Acyclovir 400 mg).	Korenromp EL, Wi T, Resch S, Stover J, Broutet N. Costing of National STI Program Implementation for the Global STI Control Strategy for the Health Sector, 2016-2021. PLoS One. 2017;12(1):e0170773. Published 2017 Jan 27. doi:10.1371/journal.pone.0170773
Gonorrhea	$11.38	Average cost per treatment across LMICs (Ceftriaxone 250 mg)	Korenromp EL, Wi T, Resch S, Stover J, Broutet N. Costing of National STI Program Implementation for the Global STI Control Strategy for the Health Sector, 2016-2021. PLoS One. 2017;12(1):e0170773. Published 2017 Jan 27. doi:10.1371/journal.pone.0170773

Chlamydia	$11.63	Average cost per treatment across LMICs (Azithromycine 500 mg)	Korenromp EL, Wi T, Resch S, Stover J, Broutet N. Costing of National STI Program Implementation for the Global STI Control Strategy for the Health Sector, 2016-2021. PLoS One. 2017;12(1):e0170773. Published 2017 Jan 27. doi:10.1371/journal.pone.0170773

Option 1 launches

	2019	2020	2021	2022	2023	2024	2025	2026	2027	2028	2029	2030	Total
**HIV**													
Vaccine Complex	0	0	0	0	0	0	0	1.73	0	2.49	0	0	4.22
**Malaria**													
Vaccine Complex	0	0	0	0	0	0	0	0.94	0	1.61	0	0	2.55
**TB**													
Vaccine Complex	0	0	0	0	0	0	0	1.07	0	0.3	0	0	1.37
**Pneumonia**													
Vaccine Simple	0	0	0	0	0.95	0	0.72	0	0	0.16	0	0.84	2.67
Vaccine Complex	0	0	0	0	0	0	0	0	0	0.04	0	0	0.04

*Fractional launches were rounded to the nearest whole number.

Option 2 launches

	2019	2020	2021	2022	2023	2024	2025	2026	2027	2028	2029	2030	Total
**HIV**													
Vaccine Complex	0	0	0	0	0	0	0	2	0	2	0	0	4
NCE Simple	0	0	0	0	0	0	0.9	0	0.5	0	0.3	0	1.7
NCE Complex	0	0	0	0	0	0	0.2	0	0.1	0	0	0	0.3
Drug Complex	0	0	0	0	0.28	0	0	0	0.1	0	1.4	1.4	3.2
Biologic Complex	0	0	0	0	0	0	0	0.9	0	2	0	0	2.4
Diagnostic Assay	0	0	0	3	3	1.5	0	0	0	0	0	0	7.5
Diagnostic Platform	0	0	0	0	1	0	1.5	0	0	0	0	0	2.5
**Malaria**													
Vaccine Complex	0	0	0	0	0	0	0	1	0	2	0	0	2.5
NCE Simple	0	0	0	0	0	0	1	0	0.4	0	0.5	0	1.9
NCE Complex	0	0	0	0	0	0	0.3	0	0.1	0	0	0.2	0.6
Drug Simple	0	0	0	0	2.56	0	3.5	3	3.4	3	3.2	0	19
Drug Complex	0	0	0	0	0.35	0	0	0	0	0	1.4	1.2	3

Biologic Complex	0	0	0	0	0	0	0	0	0	0	0	0	0
Diagnostic Assay	0	0	0	6	2	2.1	0	0	0	0	0	0	10
Diagnostic Platform	0	0	0	0	4	0	3.9	0	0	0	0	0	7.9
**TB**													
Vaccine Complex	0	0	0	0	0	0	0	1.1	0	1	0	0	1.6
NCE Simple	0	0	0	0	0	0	0.3	0	0.2	0	0.5	0	1
NCE Complex	0	0	0	0	0	0	0.6	0	0.1	0	0	0.3	1
Drug Simple	0	0	0	0	1.88	0	3.4	2.8	3	3	3.2	0	18
Biologic Simple	0	0	0	0	0	0	0	0	0	0	0	1.8	2
Diagnostic Assay	0	0	0	13	6	7.9	0	0	0	0	0	0	27
Diagnostic Platform	0	0	0	0	2	0	5.2	0	0	0	0	0	7.2
**Pneumonia**													
Vaccine Simple	0	0	0	0	1.04	0	0.7	0	0	0	0	0.9	2.8
Vaccine Complex	0	0	0	0	0	0	0	0	0	0	0	0	0.1
**Chagas**													
Vaccine Simple	0	0	0	0	0	0	0	0	0	0	0	0.9	1
Vaccine Complex	0	0	0	0	0	0	0	0	0	0	0	0	0
NCE Simple	0	0	0	0	0	0	0.3	0	0	0	0	0	0.3
Drug Simple	0	0	0	0	0.35	0	3	3.3	3.1	3	3	0	16
Diagnostic Assay	0	0	0	1	0	0.5	0	0	0	0	0	0	1.5
Diagnostic Platform	0	0	0	0	1	0	0	0	0	0	0	0	1
**Schisto**													
Vaccine Simple	0	0	0	0	0	0	0	0	0	0	0	0.9	1.3
Vaccine Complex	0	0	0	0	0	0	0	0	0	0	0	0	0
Drug Complex	0	0	0	0	0.48	0	0	0	0	0	1.4	1.3	3.1
**Leish**													
Vaccine Simple	0	0	0	0	0	0	0.2	0	0	0	0	1	1.4

Vaccine Complex	0	0	0	0	0	0	0	0.2	0	0	0	0	0.2
NCE Simple	0	0	0	0	0	0	0	0	0.4	0	0	0	0.4
NCE Complex	0	0	0	0	0	0	0	0	0.1	0	0	0	0.2
Biologic Complex	0	0	0	0	0	0	0	0	0	0	0	0	0
Diagnostic Assay	0	0	0	2	2	0	0	0	0	0	0	0	4
**Dengue**													
NCE Simple	0	0	0	0	0	0	0	0	0.1	0	0	0	0.1
NCE Complex	0	0	0	0	0	0	0	0	0	0	0	0	0
Biologic Complex	0	0	0	0	0	0	0	0	0	0	0	0	0
**Leprosy**													
Vaccine Complex	0	0	0	0	0	0	0	0.1	0	0	0	0	0.1
Drug Complex	0	0	0	0	0.29	0	0	0	0	0	1.3	1.5	3.1

*Fractional launches were rounded to the nearest whole number.

Option 3 launches.

	2019	2020	2021	2022	2023	2024	2025	2026	2027	2028	2029	2030	Total
**HIV**													
Vaccine Complex	0	0	0	0	0	0	0	2.03	0	2	0	0	4
NCE Simple	0	0	0	0	0	0	0.92	0	0.5	0	0.3	0	1.7
NCE Complex	0	0	0	0	0	0	0.17	0	0.14	0	0	0	0.3
Drug Complex	0	0	0	0	0.28	0	0	0	0.13	0	1.41	1.42	3.2
Biologic Complex	0	0	0	0	0	0	0	0.85	0	1.6	0	0	2.4
Diagnostic Assay	0	0	0	3	3	1.48	0	0	0	0	0	0	7.5
Diagnostic Platform	0	0	0	0	1	0	1.47	0	0	0	0	0	2.5
**Malaria**													
Vaccine Complex	0	0	0	0	0	0	0	0.98	0	1.6	0	0	2.5
NCE Simple	0	0	0	0	0	0	0.99	0	0.41	0	0.48	0	1.9
NCE Complex	0	0	0	0	0	0	0.27	0	0.13	0	0	0.16	0.6
Drug Simple	0	0	0	0	2.56	0	3.51	2.99	3.42	3.3	3.2	0	19
Drug Complex	0	0	0	0	0.35	0	0	0	0	0	1.42	1.22	3
Biologic Complex	0	0	0	0	0	0	0	0	0	0	0	0	0
Diagnostic Assay	0	0	0	6	2	2.1	0	0	0	0	0	0	10
Diagnostic Platform	0	0	0	0	4	0	3.89	0	0	0	0	0	7.9

**TB**													
Vaccine Complex	0	0	0	0	0	0	0	1.1	0	0.5	0	0	1.6
NCE Simple	0	0	0	0	0	0	0.33	0	0.2	0	0.46	0	1
NCE Complex	0	0	0	0	0	0	0.55	0	0.12	0	0	0.28	1
Drug Simple	0	0	0	0	1.88	0	3.42	2.82	2.96	3.4	3.21	0	18
Biologic Simple	0	0	0	0	0	0	0	0	0	0.2	0	1.77	2
Diagnostic Assay	0	0	0	13	6	7.91	0	0	0	0	0	0	27
Diagnostic Platform	0	0	0	0	2	0	5.17	0	0	0	0	0	7.2
**Pneumonia**													
Vaccine Simple	0	0	0	0	1.04	0	0.66	0	0	0.2	0	0.85	2.8
Vaccine Complex	0	0	0	0	0	0	0	0	0	0.1	0	0	0.1
**Chagas**													
Vaccine Simple	0	0	0	0	0	0	0	0	0	0.1	0	0.92	1
Vaccine Complex	0	0	0	0	0	0	0	0	0	0	0	0	0
NCE Simple	0	0	0	0	0	0	0.29	0	0	0	0	0	0.3
Drug Simple	0	0	0	0	0.35	0	2.96	3.28	3.08	3	2.95	0	16
Diagnostic Assay	0	0	0	1	0	0.49	0	0	0	0	0	0	1.5
Diagnostic Platform	0	0	0	0	1	0	0	0	0	0	0	0	1
**Schisto**													
Vaccine Simple	0	0	0	0	0	0	0	0	0	0.4	0	0.89	1.3
Vaccine Complex	0	0	0	0	0	0	0	0	0	0	0	0	0
Drug Complex	0	0	0	0	0.48	0	0	0	0	0	1.35	1.26	3.1
**Leish**													
Vaccine Simple	0	0	0	0	0	0	0.22	0	0	0.2	0	0.96	1.4
Vaccine Complex	0	0	0	0	0	0	0	0.15	0	0	0	0	0.2
NCE Simple	0	0	0	0	0	0	0	0	0.35	0	0	0	0.4
NCE Complex	0	0	0	0	0	0	0	0	0.11	0	0	0.04	0.2
Biologic Complex	0	0	0	0	0	0	0	0	0	0	0	0	0
Diagnostic Assay	0	0	0	2	2	0	0	0	0	0	0	0	4
**Dengue**													
NCE Simple	0	0	0	0	0	0	0	0	0.13	0	0	0	0.1
NCE Complex	0	0	0	0	0	0	0	0	0	0	0	0.04	0
Biologic Complex	0	0	0	0	0	0	0	0	0	0	0	0	0
**Leprosy**													
Vaccine Complex	0	0	0	0	0	0	0	0.14	0	0	0	0	0.1
Drug Complex	0	0	0	0	0.29	0	0	0	0	0	1.31	1.45	3.1
**Shigella**													
Vaccine Simple	0	0	0	0	0.62	0	1.19	0	0	0.4	0	1.18	3.4

Vaccine Complex	0	0	0	0	0	0	0	0	0	0.1	0	0	0.1
**Ebola**													
Vaccine Complex	0	0	0	0	0	0	0	0.35	0	0.5	0	0	0.8
NCE Complex	0	0	0	0	0	0	0	0	0.04	0	0	0.04	0.1
Drug Simple	0	0	0	0	1.21	0	0	0	0	0	0	0	1.2
Biologic Simple	0	0	0	0	0	0	0.17	0	0	0.6	0	0	0.8
Biologic Complex	0	0	0	0	0	0	0	0	0	0.1	0	0	0.1
Diagnostic Assay	0	0	0	12	1	0	0	0	0	0	0	0	13
**Hepatitis C**													
Vaccine Simple	0	0	0	0	0	0	0.25	0	0	0.1	0	0.86	1.2
Diagnostic Assay	0	0	0	4	0	0	0	0	0	0	0	0	4
**ETEC**													
Vaccine Simple	0	0	0	0	0.93	0	0	0	0	0.2	0	0.97	2.1
Vaccine Complex	0	0	0	0	0	0	0	0	0	0	0	0	0
Non-Typhoidal Salmonella													
Vaccine Simple	0	0	0	0	0	0	0	0	0	0.2	0	0.81	1
Vaccine Complex	0	0	0	0	0	0	0	0	0	0	0	0	0
**HAT**													
NCE Simple	0	0	0	0	0	0	0.31	0	0	0	0	0	0.3
Diagnostic Assay	0	0	0	0	0	0	0	0	0	0	0	0	0
**Onchocerciasis**													
Vaccine Simple	0	0	0	0	0	0	0	0	0	0.3	0	1.04	1.4
Drug Simple	0	0	0	0	0.26	0	3.19	2.99	2.92	3.1	3.09	0	16
Drug Complex	0	0	0	0	0	0.32	0	0	0	0	1.26	1.22	2.8
**Cholera**													
NCE Complex	0	0	0	0	0	0	0	0	0	0	0	0.03	0
**Hookworm**													
Vaccine Simple	0	0	0	0	0.38	0	0.15	0	0	0	0	0.9	1.4
**Meningitis**													
Vaccine Simple	0	0	0	0	0.32	0	0.52	0	0	0	0	0.81	1.7
Diagnostic Assay	0	0	0	0	1	0	0	0	0	0	0	0	1
Diagnostic Platform	0	0	0	0	0	0	1.43	0	0	0	0	0	1.4
**Rheumatic Fever**													
Vaccine Simple	0	0	0	0	0	0	0.4	0	0	0.3	0	0.74	1.4
**Multiple Diarrheal Diseases**													
Vaccine Complex	0	0	0	0	0	0	0	0	0	0	0	0	0
**Buruli Ulcer**													

Vaccine Complex	0	0	0	0	0	0	0	0	0	0	0	0	0
Drug Simple	0	0	0	0	0.24	0	2.97	3.02	3.05	3	2.91	0	15
Diagnostic Assay	0	0	0	3	1	0	0	0	0	0	0	0	4
**Trachoma**													
Vaccine Simple	0	0	0	0	0	0	0	0	0	0.1	0	0.94	1
**Typhoid**													
Vaccine Simple	0	0	0	0	1.36	0	0.18	0	0	0.3	0	0.87	2.7
Vaccine Complex	0	0	0	0	0	0	0	0	0	0	0	0	0
**Cryptosporidiosis**													
NCE Complex	0	0	0	0	0	0	0	0	0	0	0	0.02	0
**Multiple Salmonella Infections**													
Vaccine Simple	0	0	0	0	0	0	0	0	0	0.1	0	0.87	0.9
**Hepatitis B**													
NCE Simple	0	0	0	0	0	0	0.55	0	0.5	0	0	0	1.1
NCE Complex	0	0	0	0	0	0	0.09	0	0	0	0	0	0.1
Drug Simple	0	0	0	0	0.22	0	3.39	2.91	2.95	3.2	2.9	0	16
**Chlamydia**													
Diagnostic Assay	0	0	0	0	5	0	0	0	0	0	0	0	5
**Gonorrhea**													
Vaccine Simple	0	0	0	0	0	0	0	0	0	0.7	0	0.88	1.6
Diagnostic Assay	0	0	0	0	4	0	0	0	0	0	0	0	4
**Herpes**													
Vaccine Simple	0	0	0	0	0.33	0	0.26	0	0	0.3	0	0.83	1.7
Vaccine Complex	0	0	0	0	0	0	0	0.25	0	0	0	0	0.3

*Fractional launches were rounded to the nearest whole number.

Option 1 DALYs and treatment costs averted by disease type

	DALYs Averted	Treatment Costs Averted (2020 USD)
**HIV**	79,596,831	1,568,560,129
**TB**	56,181,690	27,822,381,922
**Malaria**	55,086,005	37,183,762,020
**Pneumonia**	324,915,651	171,806,522,677

*Negative values indicate that no treatment costs were averted. For some diseases, vaccines launched so late in the time horizon that the cost of additional cases treated due to new drugs and diagnostics was not offset by treatment costs averted through the prevention of cases by new vaccines.

Option 2 DALYs and treatment costs averted by disease type

	DALYs Averted	Treatment Costs Averted (2020 USD)
**HIV**	153,888,052	1,043,870,886
**Tuberculosis**	95,455,154	11,871,114,183
**Malaria**	98,549,522	12,537,143,191
**Pneumonia**	324,915,651	171,806,522,677
**Chagas disease**	92,018	30,088,423
**Schistosomiasis**	582,222	75,190,853
**Leishmaniasis**	348,940	24,135,705
**Dengue**	0	0
**Leprosy**	69,757	-5,601,654

*Negative values indicate that no treatment costs were averted. For some diseases, vaccines launched so late in the time horizon that the cost of additional cases treated due to new drugs and diagnostics was not offset by treatment costs averted through the prevention of cases by new vaccines.

Option 3 DALYs and treatment costs averted by disease type

	DALYs Averted	Treatment Costs Averted (2020 USD)
**HIV**	150,475,217	871,887,141
**Tuberculosis**	93,761,772	12,558,896,369
**Malaria**	97,139,975	13,336,447,404
**Pneumonia**	324,915,651	171,806,522,677
**Chagas disease**	91,972	30,104,961
**Schistosomiasis**	582,222	75,190,853
**Leishmaniasis**	332,819	28,621,715
**Dengue**	0	0
**Leprosy**	69,748	-5,601,654
**Shigellosis**	34,056,704	1,343,250,261
**Ebola**	561,123	-2,336,003
**Hepatitis C**	15,774,595	4,082,223,640
**Enterotoxigenic E.coli (ETEC)**	7,213,223	36,591,299,264

**Non-typhoidal Salmonella (NTS)**	790,757	7,393,480
**HAT**	25,255	-1,158,072
**Onchocerciasis**	4,218,733	-12,046,045
**Cholera**	0	0
**Hookworm**	14,485,009	84,444,268
**Meningitis**	55,370,544	25,678,455,231
**Rheumatic fever**	26,272,475	28,029,742,699
**Multiple diarrhoeal diseases**	0	0
**Buruli Ulcer**	4,289	-21,864,931
**Trachoma**	1,306,091	47,496,306
**Typhoid & paratyphoid**	17,937,506	1,346,185,882
**Cryptosporidiosis**	0	0
**Multiple salmonella infections**	3,098,402	356,969,612
**Hepatits B**	175,477,068	-2,690,929,207
**Herpes Simplex-2**	7,874,463	2,856,907,817
**Gonorrhea**	536,387	1,749,052,385
**Chlamydia**	800,190	-1,477,227,623

*Negative values indicate that no treatment costs were averted. For some diseases, vaccines launched so late in the time horizon that the cost of additional cases treated due to new drugs and diagnostics was not offset by treatment costs averted through the prevention of cases by new vaccines.

Cost-effectiveness and ICERs for each design option

	Net cost (2020 USD billions)		DALYs averted over 10 years (millions)	Cost per DALY averted (2020 USD)		Deaths averted over 10 years (millions)	Cost per death averted (2020 USD)	
	Societal	Investor		Societal	Investor		Societal	Investor
Option 1	$43.07	$2.80	516	$84	$5.43	18.4	$2341	$152
Option 2	$50.81	$9.84	674	$75	$14.60	22.9	$2217	$429
Option 3	$117.64	$18.61	1,030	$114	$18.01	26.91	$4,371	$692
ICER (2 vs. 1)				$48.94			$1,714	
ICER (3 vs. 2)				$186			$16,715	

Schematic of discrete event simulation for design option 1, built in SimEvents (MATLAB R2020a)

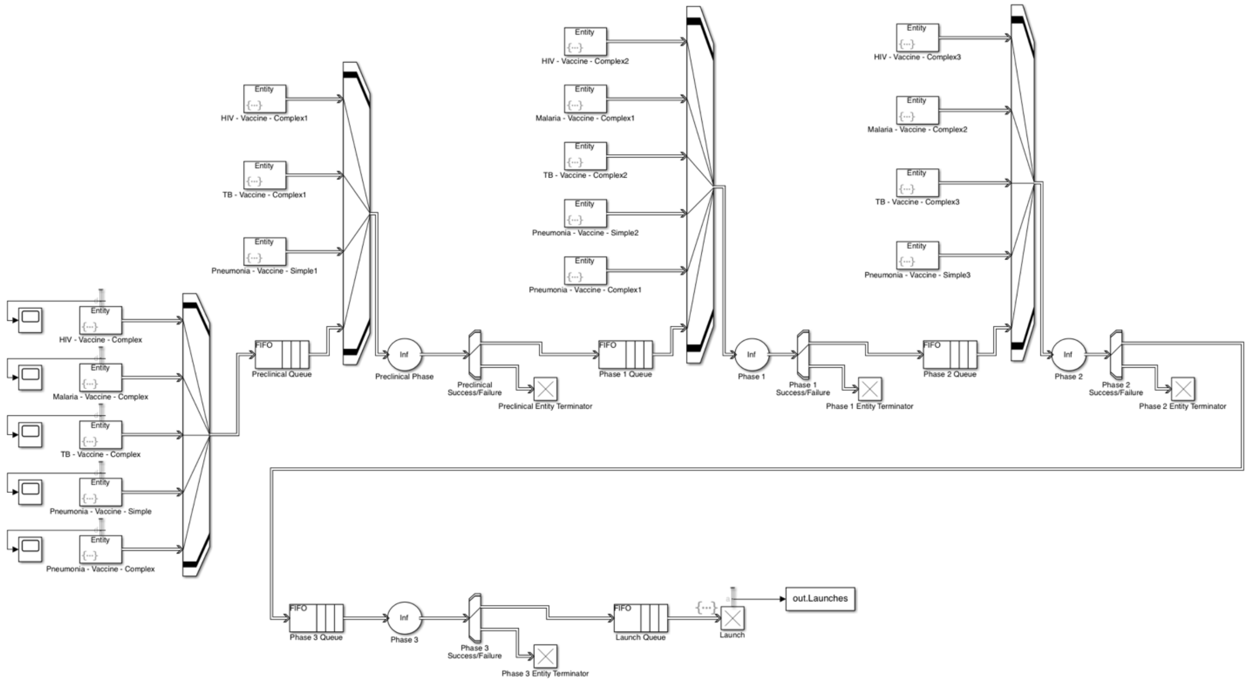

PRND vaccine, drug, and diagnostic early-stage product portfolios as of August 31, 2019.

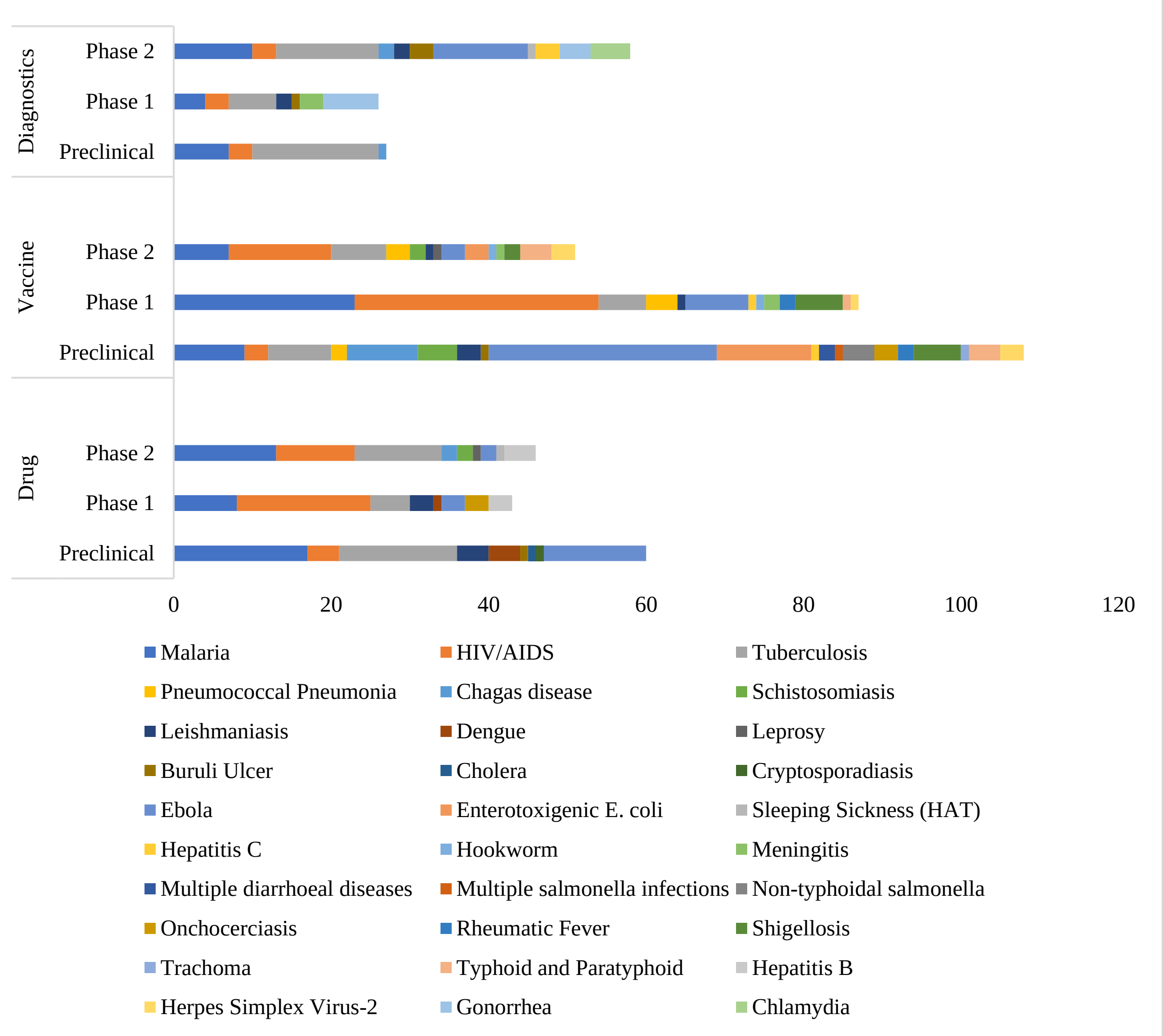

This is representative of product candidates included in Option 3 (entire portfolio). Product candidates for Options 1 and 2 are subsets of this portfolio. Note that products for multiple diarrheal disease and multiple salmonella infections are pre-clinical vaccine platforms for multiple organisms.
